# Improving Access to Refractive Services in Adults: A Health Examination Center-Based Model

**DOI:** 10.3389/fmed.2021.753257

**Published:** 2021-10-28

**Authors:** Haishuang Lin, Jing Sun, Nathan Congdon, Meiping Xu, Shanshan Liu, Yuanbo Liang, Hailin Wang, Shaodan Zhang

**Affiliations:** ^1^Department of Glaucoma, Eye Hospital and School of Ophthalmology and Optometry, Wenzhou Medical University, Zhejiang, China; ^2^National Clinical Research Center for Ocular Diseases, Zhejiang, China; ^3^Glaucoma Research Institute of Wenzhou Medical University, Zhejiang, China; ^4^Shenyang Key Lab of Ophthalmology, Department of Ophthalmology, The Fourth Peoples' Hospital of Shenyang, Liaoning, China; ^5^Centre for Public Health, Queen's University Belfast, Belfast, United Kingdom; ^6^Zhongshan Ophthalmic Center, Sun Yat-sen University, Guangzhou, China

**Keywords:** uncorrected refractive error, spectacle coverage, opportunistic screening, health examination center, refractive service

## Abstract

**Purpose:** To assess the potential of a health examination center-based screening model in improving service for uncorrected refractive error.

**Methods:** Individuals aged ≥18 years undergoing the routine physical examinations at a tertiary hospital in the northeast China were invited. Presenting visual acuity, noncycloplegic autorefraction, noncontact tonometry, fundus photography, and slit-lamp examination were performed. Refractive error was defined as having spherical equivalent ≤ -0.75 D or ≥ +1 D and uncorrected refractive error was considered as refractive error combined with presenting visual acuity < 6/12 in the better eye. Costs for the screening were assessed.

**Results:** A total of 5,284 participants (61 ± 14 years) were included. The overall prevalence of myopia and hyperopia was 38.7% (95% CI, 37.4–40.0%) and 23.5% (95% CI, 22.3–24.6%), respectively. The prevalence of uncorrected refractive error was 7.85% (95% CI, 7.13–8.58%). Women (*p* < 0.001 and *p* = 0.003), those with age ≥ 70 years (*p* < 0.001 and *p* = 0.003), and myopia (*p* < 0.001 and *p* < 0.001) were at higher risk of uncorrected refractive error and uncorrected refractive error-related visual impairment. Spectacle coverage rate was 70.6% (95% CI, 68.2–73.0%). The cost to identify a single case of refractive error and uncorrected refractive error was US$3.2 and US$25.2, respectively.

**Conclusion:** The prevalence of uncorrected refractive error is high in the urban Chinese adults. Health examination center-based refractive error screening is able to provide an efficient and low-cost model to improve the refractive services in China.

## Introduction

Uncorrected refractive error (URE), predominantly myopia, is the most common cause of moderate and severe visual impairment and the second leading cause of blindness globally, imposing a significant public health burden to the society (1, 2). Despite the relatively low cost of refractive correction, such as spectacles, the prevalence of URE remains high (3, 4). The main barrier keeping the affected adults from seeking refractive services is the absence of convenient and low-cost access to the healthcare delivery system (5).

China is one of the countries facing the greatest burden of refractive error (RE) (6). The prevalence of myopia in Chinese adults ranges from 21.1 to 62.9% (7–11). Meanwhile, China is experiencing the challenge of the high and growing prevalence of myopia among children and young adults, which is leading, in turn, to a growing burden of high myopia in adults (12). Refractive service is not well established and even absent in some underserved regions in China. Reorganizing eye care to the coexist established health services may be a way forward.

Health examination centers are well-established public health delivery system in China, which provide screening tests for early detection of specific diseases or risk factors among the population at large. People come to these centers for a general evaluation of their health status, either by the individual or organized by their employers. There are nearly 10,000 health examination centers across China covering a population of 700 million (13). These centers provide a unique platform for the screening of vision-threatening eye diseases. In this study, we report the efficacy of health examination center-based RE screening and referral among adults aged ≥18 years.

## Materials and Methods

This single-center, cross-sectional study was approved by the Ethics Committee of the Fourth People's Hospital of Shenyang and was conducted according to the tenets of the Declaration of Helsinki. The Committee determined that informed consent was not required, as data were collected in deidentified fashion and used for the purposes of health service monitoring.

### Ocular Examinations

All the participants aged ≥18 years presenting to the health examination center of the Fourth People's Hospital of Shenyang from March 1 to April 30, 2017 were invited to attend. Three trained nonmedical staff conducted the ocular examinations including assessment of presenting visual acuity (PVA) (uncorrected if the participant did not own spectacles and with distance spectacles if worn), noncontact pneumotonometry (CT−1P Computerized Tonometer, Topcon Ltd., Tokyo, Japan), noncycloplegic autorefraction (ARK-510A, Nidek Co., Ltd., Tokyo, Japan), and nonmydriatic fundus photography (Canon CX-1, Tokyo, Japan). Only staffs achieving accuracy over 95% in PVA tests during the training phase were qualified for further screening. Fundus photographs were evaluated independently by two glaucoma specialists (SDZ and YBL). For autorefraction, three consecutive readings of sphere, cylinder, and axis of each eye were taken and the mean spherical equivalent (SE) (spherical power + 12 ^*^ cylinder power) was used for analysis. Participants with PVA <6/12, intraocular pressure (IOP) ≥24 mm Hg, obvious lens opacity, or abnormalities on fundus photography in either eye were referred to the ophthalmology outpatient clinics.

### Diagnosis of Refractive Error and Uncorrected Refractive Error

Refractive error was categorized as follows by using the data from the better-seeing eye: myopia as SE ≤ −0.75 D, low myopia SE as ≤ −0.75 D to > −3 D, moderate myopia as SE ≤ −3 D to > −6 D, high myopia as SE ≤ −6 D, hyperopia as SE ≥ +1 D, and high hyperopia SE ≥ +3 D. URE was defined as RE with PVA < 6/12 in the better-seeing eye. URE-related visual impairment was defined as URE with PVA < 6/18 in the better-seeing eye (14). Participants with obvious macular or vascular abnormalities on fundus photography were excluded for the further URE-related analysis.

### Need for Spectacles

The need for spectacles was categorized as either “met” or “unmet.” “Met need” describes the number of the participants with RE as defined above achieving corrected visual acuity (VA) ≥ 6/12 in the better-seeing eye with current distance spectacles. “Unmet need” was defined as the number of the participants who did not achieve VA ≥ 6/12 with current spectacles or did not have any spectacles at all. Since the same criteria were used in this study, the “unmet need” for spectacles was equal to the URE. Spectacle coverage was defined as: met need/(met need + unmet need). Participants with unmet needs for spectacles were given a detailed explanation of their refractive condition and referred to an optometry clinic for treatment. Telephonic interviews were conducted to assess the adherence of the refraction correction in those with URE.

### Costs of Screening

The costs of the screening were calculated by using a healthcare system perspective including personnel, equipment, and overhead costs. Personnel costs were calculated as the cost of each provider participating in a certain activity specifically dedicated to program-related screening based on the 2017 mean salary catalog of Liaoning province from the National Bureau of Statistics of China (15). Costs of the equipments including VA charts, tonometer, autorefractor, and fundus camera were calculated from list prices, assuming a lifespan of 5 years (16). Overhead costs including infrastructure, electricity, water, and internet links were also assessed. All the costs were given in US dollars at the average of 2017 exchange rate [1 US dollar = 6.75 Renminbi (RMB)] (15). An annual inflation rate of 2% was used to estimate the cost in 2020 based on the 2017–2020 Consumer Price Index of the National Bureau of Statistics of China (15). To account for real-world variability, sensitivity analyses were also performed as following. Overhead costs were allowed to vary from $0 (i.e., covered by the health examination center) to +20% of the base case value. Other costs were assumed to vary by ±20% of the base case value (17). The costs to identify a single case of RE and a case of URE were calculated.

### Statistical Analysis

Statistical analyses were performed by using the Statistical Package for the Social Sciences (SPSS) for Windows, version 11.0 (SPSS, Chicago, Illinois, USA). Continuous data with normal distribution were presented as mean ± SD. Data that did not follow a normal distribution were shown as median [interquartile range (IQR)]. Prevalence of RE for different ages and genders was compared by using the chi-squared test. The multivariate regression analysis was used to investigate the impact of gender, age, and RE on the rate of URE and URE-related visual impairment. *p* < 0.05 was considered as statistically significant.

## Results

### Distribution of the Refractive Status

**Figure 1:**
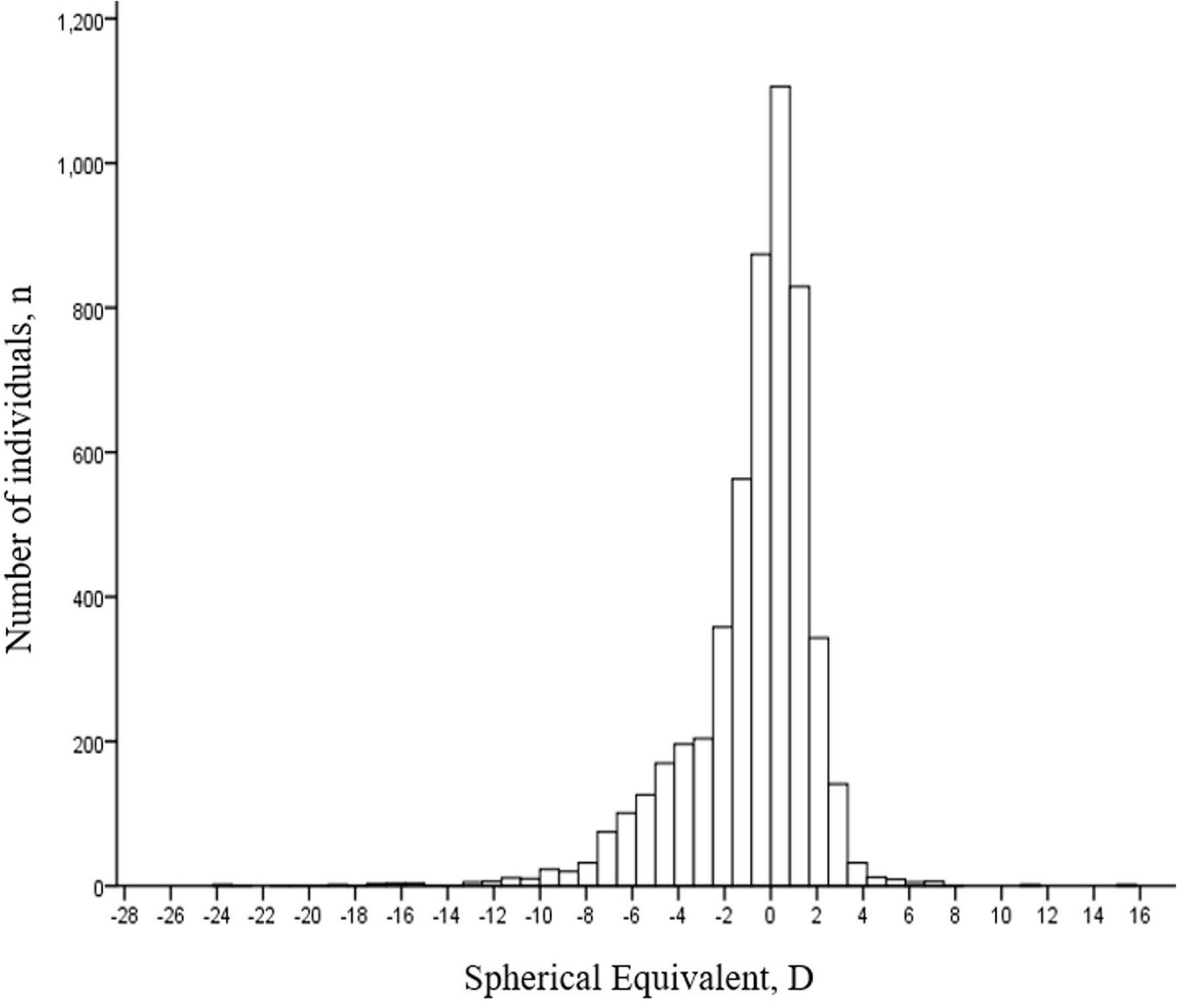
Distribution of refractive error [diopters (D)] among the screenees.

In this study, a total of 5,522 eligible adults were initially enrolled with 5,284 adults (95.7%; 95% CI, 95.2–96.2%) completing all the examinations and fulfill the criteria for analysis. The mean age of these participants was 61 ± 14 years (range, 23–96 years) and 39.4% (*n* = 2,083; 95% CI, 38.1–40.7%) were the females. The distribution of SE was leptokurtic and asymmetric with a higher frequency of the negative (myopic) refractive powers (Figure 1). The median SE was −0.25 D (IQR −1.72, 0.88). The overall prevalence of the myopia and high myopia was 38.7% (*n* = 2,046; 95% CI, 37.4–40.0%) and 5.3% (*n* = 280; 95% CI, 4.69–6.90%), respectively with an age-standardized estimate of 49.5% (95% CI, 48.1–50.8%) and 7.14% (95% CI, 6.44–7.83%), respectively. Females had a significantly higher prevalence of myopia compared to the males [47.1% (95% CI, 45.0–49.3%) vs. 33.2% (95% CI, 31.6–34.9%); χ^2^ = 103, *p* < 0.001]. The prevalence of myopia decreased steadily with increasing age from a peak of 71.8% (95% CI, 68.0–75.6%) at age <40 years to 26.5% (95% CI, 23.9–29.0%) at age ≥ 70 years (χ^2^ = 588, *p* < 0.001). The overall prevalence of hyperopia was 23.5% (*n* = 1,240; 95% CI, 22.3–24.6%) with a male preponderance [males 25.6% (95% CI, 24.1–27.2%) vs. females 20.1% (95% CI, 18.4–21.8%), χ^2^ = 21.5, *p* < 0.001]. The prevalence of high hyperopia was 2.0% (*n* = 108; 95% CI, 1.66–2.43%), higher in the females (2.4%; 95% CI, 1.7–3.0%) compared to the males (1.8%; 95% CI, 1.4–2.3%, χ^2^ = 7.09, *p* = 0.006). The age-standardized prevalence of hyperopia and high hyperopia was 11.9% (95% CI, 11.0–12.8%) and 1.31% (95% CI, 1.00–1.61%), respectively. The prevalence of hyperopia demonstrated an age-related increase ranging from 2.6 to 11.4% (95% CI, 1.4–13.2%) in the participants <60 years to 31.6% (95% CI, 29.4–33.8%) of 60–69 years and 44.9% (95% CI, 42.1–47.8%) of ≥ 70 years (χ^2^ = 739, *p* < 0.001) (Table 1).

**Table 1:** Prevalence of refractive error (RE) by the gender and age.

		**Myopia**, ***n*** **(%)**						**Hyperopia**, ***n*** **(%)**			
	**N (%)**	**All myopia**		**Low myopia**	**Moderate myopia**	**High myopia**		**All hyperopia**		**High hyperopia**	
		**≤0.75D**		**≤−0.75D to >−3.0D**	**≤−3D to >−6.0D**	**≤−6.0D**		**≥+1.0D**		**≥+3.0D**	
**Total**	5,284	2,046 (38.7)		1,157 (21.9)	609 (11.5)	280 (5.3)		1,240 (23.5)		108 (2.0)	
**Gender**											
Male	3,201 (60.6)	1,064 (33.2)	χ^2^ = 103	636 (19.9)	301 (9.4)	127 (4.0)	χ^2^ = 10.7	821 (25.6)	χ^2^ = 21.5	59 (1.8)	χ^2^ = 7.09
Female	2,083 (39.4)	982 (47.1)	*P* < 0.001	521 (25.0)	308 (14.8)	153 (7.3)	*P* = 0.005	419 (20.1)	*P* < 0.001	49 (2.4)	*P* = 0.006
**Age**											
<40	543 (10.3)	390 (71.8)	χ^2^ = 588	171 (31.5)	154 (28.4)	65 (12.0)	χ^2^ = 62.9	21 (3.9)	χ^2^ = 739	8 (1.5)	χ^2^ = 49.9
40–49	696 (13.2)	425 (61.1)	*P* < 0.001	205 (29.5)	152 (21.8)	68 (9.8)	*P* < 0.001	18 (2.6)	*P* < 0.001	7 (1.0)	*P* < 0.001
50–59	1,143 (21.6)	468 (40.9)		293 (25.6)	120 (10.5)	55 (4.8)		130 (11.4)		9 (0.8)	
60–69	1,747 (33.1)	457 (26.2)		289 (16.5)	110 (6.3)	58 (3.3)		552 (31.6)		33 (1.9)	
≥70	1,155 (21.8)	306 (26.5)		199 (17.2)	73 (6.3)	34 (2.9)		519 (44.9)		51 (4.4)	

### Prevalence of URE and URE-Related Visual Impairment

In total, 415 individuals (202 males and 213 females, mean age 63.3 ± 15.8 years) exhibited a SE > +1 D or < −0.75 D and presenting VA <6/12 in the better-seeing eye, presenting a prevalence of 7.85% (95% CI, 7.13–8.58%) for URE. Among these participants with URE, 360 (86.7%; 95% CI, 83.5–90.0%) were unaware of their refractive status and had never been diagnosed with RE, 24 (5.8%; 95% CI, 3.5–8.0%) were wearing inappropriate spectacles, and 31 (7.5%; 95% CI, 5.9–10.0%) had been diagnosed previously, but did not fill the prescription given to them for the glasses. URE-related visual impairment was observed in 3.0% (*n* = 157; 95% CI, 2.5–3.4%) participants. Women (χ^2^ = 14.67, *p* < 0.001 and χ^2^ = 7.962, *p* = 0.003), those with age of 70 years and older (χ^2^ = 27.21, *p* < 0.001 and χ^2^ = 15.84, *p* = 0.003), and myopia (χ^2^ = 56.87, *p* < 0.001 and χ^2^ = 50.81, *p* < 0.001) are at higher risk of both URE and URE-related visual impairment (Table 2).

**Table 2:** The prevalence of uncorrected refractive error (URE) and URE-related visual impairment (VI) according to the age, gender, and type of RE.

	***N***	**URE**		**URE related VI *n* (%) [95%CI]**	
		***n* (%) [95%CI]**			
**Total**	5,284	415 (7.85) [7.13–8.58]		157 (2.97) [2.51–3.43]	
**Gender**					
Male	3,201	202 (6.31) [5.47-7.15]	χ^2^ = 14.67	73 (2.28) [1.76–2.80]	χ^2^ = 7.962
Female	2,083	213 (10.2) [9.66–12.3]	*P* < 0.001	84 (4.03) [3.19–4.88]	*P* = 0.003
**Age**					
<40	543	45 (8.29) [5.96–10.6]	χ^2^ = 27.21	17 (3.13) [1.66–4.60]	χ^2^ = 15.84
40–49	696	53 (7.61) [5.64–9.59]	*P* < 0.001	19 (2.73) [1.52–3.94]	*P* = 0.003
50–59	1,143	83 (7.26) [5.75–8.77]		33 (2.89) [1.91–3.86]	
60–69	1,747	93 (5.32) [4.27–6.38]		31 (1.77) [1.15–2.39]	
≥70	1,155	141 (12.2) [10.3–14.1]		57 (4.94) [3.68–6.19]	
**Type of refractive error**					
Myopia	2,046	328 (16.0) [14.4–17.6]	χ^2^ = 56.87	140 (6.84) [5.75–7.94]	χ^2^ = 50.81
Hyperopia	1,240	87 (7.02) [5.59–8.44]	*P* < 0.001	17 (1.37) [0.72–2.02]	*P* < 0.001

### Need and Coverage of Spectacles

Out of the 3,286 participants with RE (2,046 myopia and 1,240 hyperopia), 997 participants [526 males (52.8%; 95% CI, 49.7–55.9%) and 471 females (47.2%; 95% CI, 44.1–50.4%)] with mean age (53.3 ± 14.7 years) achieved presenting VA of 6/12 or above in the better eye with their current spectacles (met need). The unmet need was equal to the value of URE (*n* = 415) including 24 (5.8%; 95% CI, 3.5–8.0%) undercorrected and 391 (94.2%; 95% CI, 92.0–96.5%) uncorrected. Spectacle coverage rate was 70.6% [997/(997 + 415) ×100%] (95% CI, 68.2–73.0%) in the present RE population. Younger adults with age < 40 years had a peak of spectacle coverage 83.9% (95% CI, 79.6–88.3%), compared to those with age ≥ 70 years [48.5% (95% CI, 42.6–54.5%)] (χ^2^ = 103, *p* < 0.001). Participations with myopia [73.5% (95% CI, 71.0–75.9%)] had significantly higher spectacle coverage, compared to those with hyperopia [50.6% (95% CI, 43.1–58.0%)] (χ^2^ = 38.9, *p* < 0.001). demonstrated significantly higher spectacle coverage (Table 3). After the screening, 137 individuals with URE (33.0%; 95% CI, 28.5–37.6%) adhered to the referral suggestion and got their RE corrected with the spectacles.

**Table 3:** Spectacle need and coverage by the gender, age, and type of RE.

	**RE-suspects**	**RE need spectacles**		**Met need**	**Unmet need**	**Spectacle coverage**	
	***n***	***n* (%)**		***n***	***n***	**% (95%CI)**	
**Total**	3,286	1,412 (43.0)		997	415	70.6 (68.2–73.0)	
**Gender**							
Male	1,885	728 (38.6)	χ^2^ = 34.1	526	202	72.3 (69.0–75.5)	χ^2^ = 1.956
Female	1,401	684 (48.8)	*P* < 0.001	471	213	68.9 (65.4–72.3)	*P* = 0.162
**Age**							
<40	411	280 (68.1)	χ^2^ = 264	235	45	83.9 (79.6–88.3)	χ^2^ = 103
40–49	443	277 (62.5)	*P* < 0.001	224	53	80.9 (76.2–85.5)	*P* < 0.001
50–59	598	265 (44.3)		182	83	68.7 (63.1–74.3)	
60–69	1,009	316 (31.3)		223	93	70.6 (65.5–75.6)	
≥70	825	274 (33.2)		133	141	48.5 (42.6–54.5)	
**Type of refractive error**							
Myopia	2,046	1,236 (60.4)	χ^2^ = 673	908	328	73.5 (71.0–75.9)	χ^2^ = 38.9
Hyperopia	1,240	176 (14.2)	*P* < 0.001	89	87	50.6 (43.1–58.0)	*P* < 0.001

### Costs of the Screening

The total cost of this 2-month program was estimated at US$10,449 (sensitivity analysis, varying from US$6,640 to US$12,539). Staff costs comprised US$4,892 (varying from US$3,914 to US$5,871) with equipment accounting for US$3,408 (varying from US$2,726 to US$4,089) and overhead costs US$2,149 (varying from US$0, if the health examination center covers the fees, to US$2,579). The cost to identify a single case of suspected RE and URE was US$3.2 (varying from US$2.0 to US$3.8) and US$25.2 (varying from US$16.0 to US$30.2), respectively. The cost for identifying per case of URE-related visual impairment was US$66.6 (varying from US$42.3 to US$79.9) (Table 4).

**Table 4:** Costs for the screening.

**Item**	**Units**	**Costs (US$, [range])**	**Methods for calculation**
**Personnel**			
**Staff training**			
Visual acuity test	1	547 (437, 656)	[$6180 per year/12 x 0.5 month working time x 2 month screening x (100 + 2%)^3^] x (100 ± 20%)
IOP^*^ and auto-refraction	1	547 (437, 656)	[$6180 per year/12 x 0.5 month working time x 2 month screening x (100 + 2%)^3^] x (100 ± 20%)
Fundus photography	1	547 (437, 656)	[$6180 per year/12 x 0.5 month working time x 2 month screening x (100 + 2%)^3^] x (100 ± 20%)
**Ophthalmologists' time**			
Slit-lamp examination	1	902 (722, 1,082)	[$10200 per year/12 x 0.5 month working time x 2 month screening x (100 + 2%)^3^] x (100 ± 20%)
Photograph reading	2	1,804 (1,443, 2,165)	[$10200 per year/12 x 0.5 month working time x 2 month screening x 2 x (100 +2%)^3^] x (100 ± 20%)
**Secretaries' times**			
Data registration	1	547 (437, 656)	[$6180 per year/12 x 0.5 month working time x 2 month screening x (100 + 2%)^3^] x (100 ± 20%)
**Equipment for screening**			
Visual acuity chart	1	1.6 (1.3, 1.9)	[$44.4/ a life span of 5 years x2/12 x (100 + 2%)^3^] x (100 ± 20%)
Noncontact tonometer	1	524 (419, 629)	[$14814/ a life span of 5 years x2/12 x (100 + 2%)^3^] x (100 ± 20%)
Autorefractor	1	524 (419, 629)	[$14814/ a life span of 5 years x2/12 x (100 + 2%)^3^] x (100 ± 20%)
Fundus camera	1	1,834 (1,467, 2,201)	[$51852/ a life span of 5 years x2/12 x (100 + 2%)^3^] x (100 ± 20%)
Slit-lamp	1	524 (419, 629)	[$14814/ a life span of 5 years x2/12 x (100 + 2%)^3^] x (100 ± 20%)
**Overhead costs**			
Rental costs of screening clinic	1	1,573 (0, 1,887)	[$741/month x 2 months x (100 + 2%)^3^] x (0, 100 + 20%)
Electricity and water	1	314 (0, 377)	[$148/month x 2 months x (100 + 2%)^3^] x (0, 100 + 20%)
Internet system link up	1	262 (0, 314)	[$7407/ a life span of 5 years x2/12 x (100 + 2%)^3^] x (0, 100 + 20%)
**Total**		10,449 (6,640, 12,539)	

A healthcare system perspective was used for the cost assessment. All the costs are in US dollars at the average of 2017 exchange rate (1 US dollar = 6.75 RMB) and with 2020 costs estimated by using an inflation rate of 2% base on the 2017–2020 Consumer Price Index of China. Personnel costs were calculated by using the mean salary registry of the Liaoning province in 2017 based on the data from the National Bureau of Statistics of China (15). A variation of 20% was assumed for the sensitivity analysis (17, 18). Overhead costs were allowed to vary from $0 (i.e., covered by the health examination center) to +20% of the base case value. Other costs were assumed to vary by ±20% of the base case value. ^*^IOP, intraocular pressure.

## Discussion

A most recent survey on the causes of vision loss in China revealed that URE remains the major leading cause of moderate/severe vision impairment and blindness in China in the overall population (19, 20). India and China account for approximately 50% of global vision impairment and blindness due to UREs (4, 21, 22). Improving the public awareness of visual health and the unmet demand for refractive care among the affected adults are still big challenges. In this study, we reported the outcome of integrating the RE screening into the general health examination, a well-established public health delivery system in China. It possesses the advantages of easy recruitment of screenees, reductions in demand for the human resources through scale, and the lower costs for equipment and travel, providing a potentially ideal opportunity for a large population to contact the available and routine eye care service by convenience.

Detected prevalence of RE and URE greatly depends on the screening method and criteria used. During health examination, an intensive population (usually 200–400) needs to be screened within 3 to 4 h in the morning. To guarantee the confluence of the entire procedure, a quick and noninvasive refractive assessment method is arbitrarily required. Visual correction by subjective refraction test or pinhole glasses is time-consuming and not suitable for the present protocol. It has been reported that SE of autorefraction were statistically similar compared to subjective refraction and were able to provide the reasonable and repeatable estimation of RE in the adults (23). In population with a high prevalence of RE, combining the uncorrected VA and noncycloplegic autorefraction in serial order, it achieved the adequate sensitivity and specificity for RE screening (24–26). So, we utilized a combination of autorefraction and presenting VA test for the diagnosis of URE in current screening. Considering the accuracy and specificity of the autorefraction test, a strict criterion was introduced (SE ≤ −0.75 D for myopia and ≥ +1 D for hyperopia) (27, 28). In this study, we invited all the individuals of 18 years and older coming to the centers for routine health examination without sampling. The prevalence of present URE may not be comparable with that derived from the population-based studies, especially when the different diagnostic criteria were used (29–36) (Table 5). Meanwhile, the impacts of URE in the Chinese adults were usually addressed when analyzing the cause of visual impairment (19, 20, 37–39). Studies directly reporting the prevalence, distribution, and service coverage for URE among the adults in China are very limited. Although with these differences, the URE in the present urban Chinese population is quite high (7.9%; 95% CI, 7.13–8.58%). Consistent with the previous studies, we observed higher URE in the older individuals (30, 35). Females demonstrated higher prevalence of URE (10.2%; 95% CI, 9.7–12.3%) and URE-related visual impairment (4.0%; 95% CI, 3.2–4.9%) compared to the males (6.3%; 95% CI, 5.5–7.2% and 2.3%; 95% CI, 1.8–2.8%, respectively). It is consistent with the previous findings that the older and female individuals persistently bear more burden of URE than their counterparts over the past few decades (4, 40). A gender-sensitive health policy may be helpful for managing the gender inequality in global vision loss caused by URE.

**Table 5:** Prevalence of URE among the different studies.

**Country**	**Year**	**Age**	**N**	**Prevalence of URE (%)**	**Definition of refractive error and URE**
China	Present	23–96	5,284	7.85	Presenting visual acuity (PVA) <6/12, SE≥+1D or ≤ -0.75D in the better eye
Armenia	2021	51–94	485	26	PVA <6/12 but improved ≥ one line after refraction in the better eye
Australia	2020	40–92 (Indigenous)	1,738	14.5	PVA <6/12, improved ≥2 lines by correction
		50–98 (non-indigenous)	3,098	11	Improvement of ≥2 lines on the logMAR chart in one or both eyes in participants with a PVA <6/12
India	2019	≥30	Meta-analysis	10.2	PVA <6/18, ≥+0.5D ametropia, improved ≥2 lines by correction
Colombia	2019	15–96	2,886	12.5	PVA <20/40 but correctable to ≥20/40 using a pinhole
France	2019	≥78	707	38.8	PVA in the better-seeing eye improved by at least 5 letters on the ETDRS chart (≥1 line on the logMAR chart)
Iran	2019	≥ 60	3,310	8.85	Visual acuity worse than 20/40 in the better eye without correction and could achieve 20/40 or better with correction
Brazil	2014	>1	7,654	13.8	Non-corrected visual acuity >0.15 logMAR and Best corrected visual acuity ≤ 0.15 logMAR after refractive correction
British	2012	48–89	4,428	1.9	≥1 line improvement of visual acuity with pinhole-correction in the better eye in participants with LogMar presenting visual acuity (PVA) <0.3
Japan	2011	≥40	1,381	4.8	PVA <6/18 in the better eye, improved to at least 6/18 with pinhole
Singapore	2004	40–79	1,152	17.3	Improved ≥2 lines by correction
Iran	2002	5–95	4,353	4.8	≥+0.5D and ≤ -0.5D

Spectacle coverage is another index of refractive service (5, 24, 41–43). The spectacle coverage rate in the present urban adults (≥18 years, 70.6%; ≥40 years, 67.3%; ≥60 years, 60.3%) was lower than that reported in the Australians (≥40 years, 82.2–93.5%), but higher than that among the semi-rural adults in Shanghai, China (≥60 years, 44.1%), India (≥15 years, 33.1%; ≥40 years, 53.6%), Colombia (≥15 years, 50.9%), Kenya (≥50 years, 25.5%), and Nigerian (≥40 years, 4.4%) (36, 44–49). Barriers to the spectacle use mainly include economic stability of the society and individuals, limited refractive care access, and poor health awareness (50).

Inconvenience associated with wearing glasses, the uncertainty of the perceived benefit, and lack of social desirability may also contribute to the low spectacle use (51). In a community-based screening program in Baltimore, 72% of the individuals with unmet need of spectacles did not obtain eyeglasses even with a very low price (52). Similarly, in this study, only one-third of the individuals with URE adhered to the referral suggestion and got their spectacles. Strategies to improve the public awareness of visual health are of great challenge (19). Incorporating educational content may help to improve the knowledge and awareness about URE, while introducing ready-made spectacles into vision screening may help to provide a direct experience of VA improvement and increase demand for and compliance with spectacles (53, 54). Recently, we are introducing a wavefront aberration-based subjective autorefraction into this screening model. Individuals with RE can obtain a refractive prescription immediately after the examination, which may help to increase the diagnostic accuracy and improve the adherence of spectacle use among those affected individuals. In addition, an additional advantage of the present model is that the health examinations at most of the centers are repeated annually, offering the potential to improve the compliance in the unresponded suspects over time.

The cost for RE screening varies significantly among different countries and settings, depending on the capacity for service, personnel involved, the type and amount of equipment utilized, and the model used. Being integrated into a coexist healthcare system, the demand for human resources, traffic fees, and other overhead costs in the present screening model were greatly reduced. Accordingly, the cost of present screening (US$3.2 per case of RE and US$25.2 per case of URE) was significantly lower than that among school children in rural China, which reported an average cost of US$37.53, US$52.19, and US$59.14 for per case of RE detected in the teacher, optometrist, and volunteer screening model, respectively (55). Noteworthy, the cost in this study not only includes fees for the screening of RE, but also for the detection of other suspected eye diseases including glaucoma, ocular hypertension, and retinal vascular diseases. In addition, the cost of present screening will be further decreased when it is applied to a larger population.

This study has some limitations. First, presbyopia, which is the most common cause of vision impairment in older adults, was not included in this study. Given the very high prevalence of presbyopia among working-age adults, low rates of correction in China and the modest cost of near-vision testing, it seems likely that such inclusion would have reduced the total program cost per beneficiary. Second, a threshold of VA <6/12 was used when calculating the prevalence of URE and the unmet need of the spectacles. However, individuals with RE and 12/12 ≥ PVA ≥ 6/12 may also benefit from the spectacles. Refractive correction among this population will further improve their quality of vision and, therefore, augment the efficiency of the present model. Third, without a corrected VA, we are not able to distinguish all the individuals that could benefit from a refractive correction. As discussed above, we are now introducing a wavefront aberration-based subjective autorefraction into this screening model to increase the diagnostic accuracy and to provide direct prescription of the spectacles to those affected individuals.

China bears a great burden of URE-related visual impairment in adults. The model of integrating RE screening into the general health examination is efficient, inexpensive, and practical. Unlike population-based or community-based screening that was initiated by the government or public health institutes, individuals come to the health examination centers for a routine physical examination actively. It facilitates greater contact with the healthcare system among the at-risk persons. Meanwhile, this integrated model greatly decreases the costs (traffic and infrastructure) of the screening. By scaling up this eye disease screening model more widely, we can better target the very large and growing cohort of individuals in China to improve the detection of URE and other ocular diseases.

## Data Availability Statement

The raw data supporting the conclusions of this article will be made available by the authors, without undue reservation.

## Ethics Statement

The studies involving human participants were reviewed and approved by the Ethics Committee of the Fourth People's Hospital of Shenyang. Written informed consent for participation was not required for this study in accordance with the national legislation and the institutional requirements.

## Author Contributions

HL contributed to the formal analysis and the original draft writing. JS contributed to the investigation and formal analysis. NC contributed to the writing, review, and editing. MX contributed to the validation and data curation. SL contributed to the investigation and resources. YL contributed to the supervision. HW contributed to the project administration and funding acquisition. SZ contributed to the supervision, conceptualization, methodology, funding acquisition, writing, and the draft revising.

## Funding

This study was funded by National Key Research and Development Program of China (2020YFC2008200), the Basic Scientific Research Program of Wenzhou (Y20190695), Key Innovation and Guidance Program of the Eye Hospital, Wenzhou Medical University (YNZD2201903), and the Shenyang Key Technology Research and Development Project (17-230-9-03). NC was supported by the Ulverscroft Foundation (UK) and was the Research Director for the Orbis International, a nongovernmental organization providing refractive error services in China.

## Conflict of Interest

The authors declare that the research was conducted in the absence of any commercial or financial relationships that could be construed as a potential conflict of interest. The reviewer CL declared a shared affiliation with one of the authors, NC, to the handling editor at time of review.

## Publisher's Note

All claims expressed in this article are solely those of the authors and do not necessarily represent those of their affiliated organizations, or those of the publisher, the editors and the reviewers. Any product that may be evaluated in this article, or claim that may be made by its manufacturer, is not guaranteed or endorsed by the publisher.
